# Relocatable, Automated Cost-Benefit Analysis for Marine Sensor Network Design

**DOI:** 10.3390/s120302874

**Published:** 2012-03-02

**Authors:** Claire D’Este, Paulo de Souza, Chris Sharman, Simon Allen

**Affiliations:** 1 Tasmanian ICT Centre, CSIRO, Castray Esplanade, Hobart, TAS 7000, Australia; E-Mails: paulo.desouza@csiro.au (P.S.); chris.sharman@csiro.au (C.S.); 2 CSIRO Marine and Atmospheric Research, Castray Esplanade, Hobart, TAS 7000, Australia; E-Mail: simon.allen@csiro.au

**Keywords:** sensor network design, marine monitoring

## Abstract

When designing sensor networks, we need to ensure they produce representative and relevant data, but this must be offset by the financial cost of placing sensors. We describe a novel automated method for generating and combining cost and benefit values to decide on the best sensor locations using information about the specific constraints available in most coastal locations. Costs in maintenance, negotiation, equipment, exposure and communication are estimated using hydrodynamic models and Electronic Navigation Charts. Benefits in maximum coverage and reducing overall error are also determined using model output. This method demonstrates equivalent accuracy at predicting the whole system to expert-chosen locations, whilst significantly reducing the estimated costs.

## 1. Introduction

Sensor networks can provide useful data for small organisations with low budgets, however there is still much work required to make low-cost marine monitoring possible. The Tasmanian Marine Analysis Network (TasMAN) project has focused on the development of low-cost sensor nodes [1] and has included methods for assuring data quality when using cheaper and less reliable sensors [2]. The work presented in this paper reduces costs at the network planning stage.

The TasMAN project has six long-term, permanent sensor nodes and is manufacturing another 50 over the coming year. As a part of this process we are performing a cost-benefit analysis for sensor node placement. We have identified the potential benefits of a monitoring location as providing useful, representative data for our stakeholders, and identified the costs as construction, deployment and maintenance of the sensor nodes. The aim is to develop a sensor network design method that is automated, scalable, efficient, relocatable, reproducible and cost efficient. The cost and benefit scores are derived from information available in Electronic Navigational Charts (ENCs) and from a relocatable hydrodynamic model.

We could use domain knowledge to decide upon sensor locations, but those deploying the sensor network will not always be domain experts, nor have experts readily available. However, we may have access to domain knowledge in the form of physical models that overlap with areas we would like to place sensor networks. Environmental scientists are developing complex and reliable physical models in disciplines such as hydrology [3], biogeochemistry [4], and meteorology [5]. Our project focuses on the marine environment of Australia and we are fortunate enough to have a detailed hydrodynamic model for several regions and years [6]. The model is relocatable and in the process of migrating to an open source solution, which will allow it to be applied at new locations without cost.

This paper describes the automated design of a sensor network considering cost-benefit analysis. We evaluate the value of placing a node at each grid point of the model. The sensor placement solution described in this paper is called Automated Cost-Benefit Analysis (ACBA).

### Related Work

There are multiple approaches to the sensor location problem. Choosing locations can be automated with a random function, or a function with a minimum distance threshold. We can also use more elaborate techniques such as k-median to ensure optimal spatial coverage of an area [7]. There are also methods for reducing the costs of communicating between nodes [8,9].

If we have prior knowledge about an area of interest, such as a model, we can try to find the optimal model-based design. Fixed observing systems have been designed and evaluated with the aid of physical models from the 1980s [10] until now [11]. However, these methods have not integrated the associated costs and practicalities into the design.

Frolov *et al*. [12] use observations from an existing network to try and redesign it. Optimal model-based approaches use techniques such as simulated annealing to find the global minimum of variance or error. Such techniques are extremely computationally expensive, sometimes taking days to run, still include a degree of randomness and there is no guarantee they will adequately cover regions with disparate local behaviour. For example, in our area of interest South-East Tasmania there are regions upriver where salt and fresh water mix. These areas are small in size and will therefore have little effect on the average error or variance of the entire region. However, these areas are crucial to our understanding of the region and are of particular concern in estuary systems. For low resolution oceanic models optimisation techniques may be more appropriate, but these can still be factored into the automated cost-benefit analysis described in this paper. If an optimisation approach is feasible for the domain then this can be included as a benefit with the optimal location receiving the highest score.

These past approaches have not attempted to combine multiple, contributing cost factors to create an overall solution. ACBA provides this in an automated and easily relocatable method.

## 2. System Specifications

The following section outlines the data sources for deriving the cost-benefit scores, and describes the sensor nodes that are under consideration for placement at each potential location.

### 2.1. Sensor Nodes

The TasMAN low-cost sensor nodes are equipped with temperature, conductivity, and pressure sensors at different depths in the water column. The node may carry optional dissolved oxygen and fluorometer sensors. They are solar powered and communicate independently via 3G modems and do not require sink nodes or gateways [1]. The sensor nodes are attached to buoys or existing infrastructure, such as marinas or channel markers. With a base sensor payload the cost of each node is estimated at US$1,600 for a buoy based node and US$600 for a node attached to infrastructure. Costs for sensors are variable. An example 10m sensor string with temperature sensors spaced at 1m intervals, two conductivity sensors and one pressure sensor would cost about US$750. The real-time data is integrated with models for forecasting river conditions and made available through web and mobile applications for science, government and industry [13].

### 2.2. Electronic Navigational Charts

The ENC charts are vector datasets in a format called S-57. These charts are usually used to support navigation in vessels. Charts are available worldwide for most populated coastal areas.

### 2.3. Near Real-Time Hydrodynamic Model

**Figure 1.**
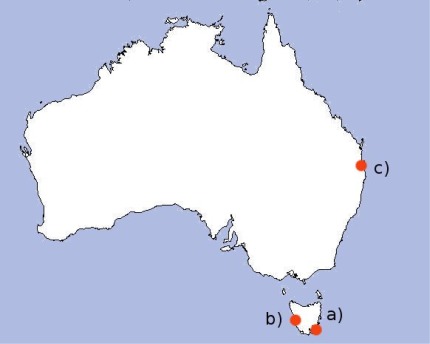
Regions with available hydrodynamic models explored in this paper: (**a**) South East Tasmania; (**b**) Macquarie Harbour; and (**c**) Moreton Bay.

The hydrodynamic model is based on Herzfeld’s general purpose model for estuaries to regional ocean domains [14]. The main model is for South-East Tasmania (43.1°S, 147.5°E), but models are also applied at Macquarie Harbour (42.3°S, 145.4°E), Moreton Bay (27.2°S, 153.2°E), and the Great Barrier Reef (20.1°S, 149.9°E). The three regions explored in this paper are shown in Figure 1. The model provides three-dimensional distributions of temperature, salinity, current velocity, density, passive tracers, mixing coefficients and sea level. From inputs such as wind, pressure, surface heat and tides, the model calculates momentum, continuity, and conservation of heat and salt. The model is based on the primitive equations and employs the hydrostatic and Boussinesq assumptions on currents [15]. The grid itself is non-uniform and curvilinear with grid spacing between approximately 200 m and 800 m. There is higher resolution upriver and in coastal regions, and relatively low resolution further out to sea.

The model output is stored in NetCDF (Network Common Data Form) machine-independent format for representing scientific data [16]. We use the grid format from the NetCDF files as potential locations. The output used to create the costs and benefits in the following experiments ranges from February 2008 to February 2010 and is in hourly intervals. Test data was extracted from model output ranging between August 2010 and December 2010. This represents over 144 million data points.

### 2.4. Glider Transects

We also take into account in the planning process that there are currently underwater vehicles repeating the same transect in South East Tasmania. The ocean glider takes multiple environmental sensor readings including; temperature, salinity, chlorophyll, dissolved oxygen. We do not want to repeat the information provided by these gliders, or potentially interfere with the missions, so model grid points that the glider regularly passes through are counted as existing sensor nodes.

## 3. Estimating Costs

The first stage is to identify the quantifiable costs associated with each potential location. In the case of the TasMAN project, much of this can be extracted from the hydrodynamic model at a high resolution given the large area, or from Electronic Navigation Charts.

For the initial area under consideration, the approximately 100 km^2^ area in South East Tasmania, the costs vary greatly. We have identified five contributing costs:

- Maintenance
- Equipment
- Exposure Factors
- Negotiation
- Communication

The following sections describe methods we have developed to automate the generation of a cost for each of these contributions at each potential location.

### 3.1. Maintenance

Sensors require regular maintenance and calibration. In marine sensor networks, this may need to be as often as monthly because of bio-fouling. As we move towards low-cost sensors and sensor nodes this is even more evident. The further a sensor node is from our base of operations, the more expensive it will be to deploy and maintain. In the case of our marine sensor network, we have the cost of boat hire; including fuel and crew. There is a cost per hour, which will increase the further we have to travel, and as we navigate further off the coast we will need to hire larger and more expensive boats. This is quantified by distances from our base.

The A* algorithm [17] is applied to the model grid cells to calculate the distance from each grid point from our base to be traversed by boat. A* is an algorithm that attempts to find the shortest path between two points, which may include points that are inaccessible - in this case we cannot pass through locations that are on land. Once A* had chosen the shortest path *P* through the grid cells then the average grid cell size of each step *p* is summed together to create a total distance. In Equation (1), *w* is the width and *h* is the height.

(1) $$\mathrm{dist}(P)=\sum_{p\in P}\frac{w_{p}+h_{p}}{2}$$

**Figure 2.**
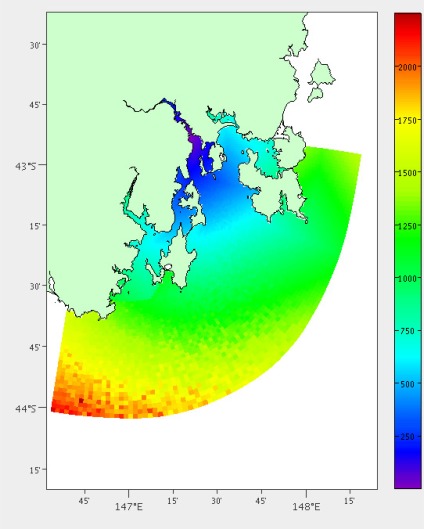
Estimated distance by boat from our laboratory in metres.

This is required because of the non-uniform grid. If we only counted the number of grid cells passed it would consider crossing the areas upriver with high resolution to have greater distance than the large grid cells out in the ocean. Figure 2 shows the results from this analysis.

At other locations it will be more efficient to drive to the waters edge and launch a boat from there, also we may be able to reach the sensor from a jetty or other infrastructure and a boat is not required. We quantify this by the distance-by-land.

**Figure 3.**
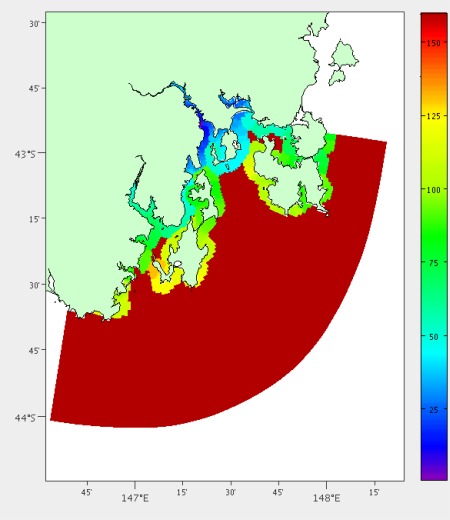
Estimated duration in minutes from our laboratory using available roads.

The distance-by-land was calculated using Google Maps Directions API [18], which determines the shortest distance by road and the expected duration of the trip. Many of the grid points were too far out to sea for this calculation to be valid and are given a maximum cost. We are more interested in the duration of the trip rather than the distance as some journeys would be through busy city areas or require waiting for a ferry in the case of points off Bruny Island (43°25′S, 147°10′E). The results from this analysis is shown in Figure 3.

**Figure 4.**
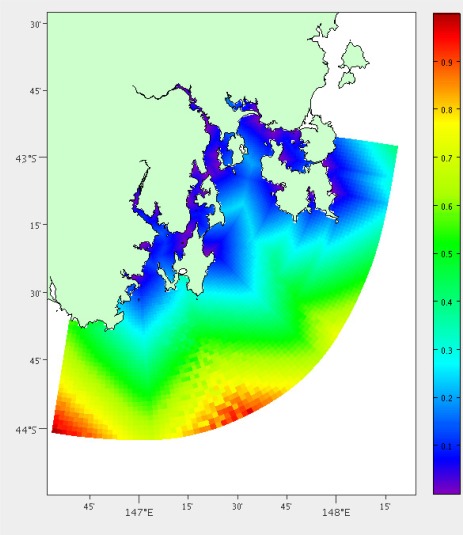
Distance from the nearest jetty in metres using A* pathfinding.

The distance-by-boat cost is also reduced based on the distance from the nearest jetty, Figure 4. Driving to a boat ramp will often be faster and save fuel. We again use A* star pathfinding to determine the distance. The coordinates of the jetties were provided by our local marine and safety authority.

### 3.2. Equipment

If we manufacture identical sensor nodes for each location than this will save on equipment costs. However, each time we have to customise a node this will incur costs in staff time and any additional parts. One of the potential factors for customisation in the TasMAN sensor nodes is based on the mooring. The deeper the water the more sophisticated the anchoring equipment required and in very deep water it may not be possible to anchor at all. We have the additional constraint that we require greater than three metres of water to immerse the sensor string. This is the case because we wish to have sensors at at least two different depths at each location and there is one metre between each set of sensors.

**Figure 5.**
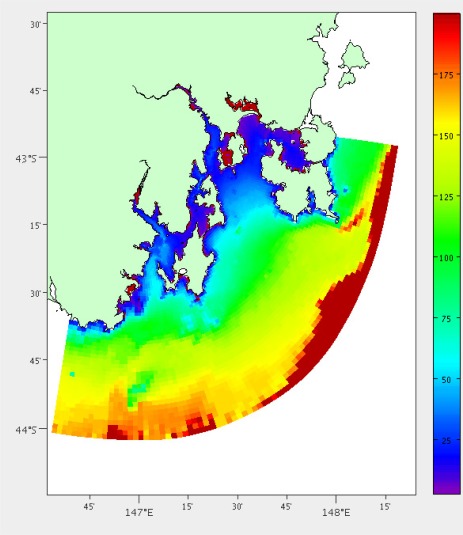
Depth cost derived from the hydrodynamic model for South East Tasmania.

Figure 5 depicts depth information for each of the grid points from the hydrodynamic model. The cost is based on the depth of water with deeper water having higher cost. Grid points with less than three metres depth will also incur a higher cost as we would have to alter the sensor node design.

In other environments the additional equipment costs may be based on other factors such as soil type.

### 3.3. Exposure Factors

There are some locations that will have increased risk of damaging, losing or destroying the sensor nodes. If we want to reduce the potential cost of repairs and replacement than this should also be quantified. For the TasMAN project, we have to consider rough seas that might cause the sensor node to break free of anchor or be destroyed. Other domains may have to be concerned with factors such as high winds, or lightening storms. Security is another consideration of exposure. It may be possible to identify some areas as having greater risk of theft.

#### Current Velocity

**Figure 6.**
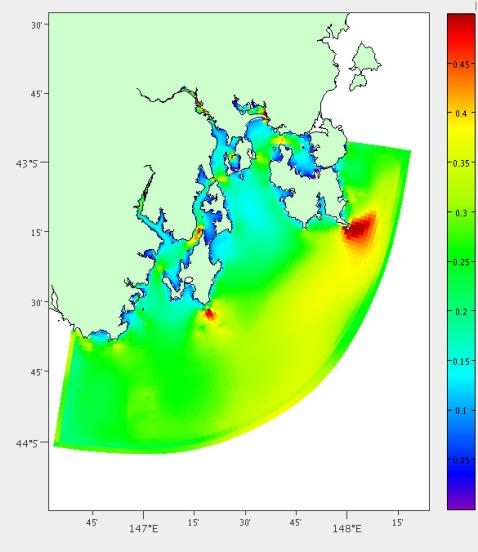
Mean current velocity in metres per second for South East Tasmania from hydrodynamic model output.

High current areas will be more difficult to deploy in, as well as having higher risk to the mooring itself. The mean of the current velocity at each location is determined using over two years of hydrodynamic model data for each of the grid points. The results can be viewed in Figure 6.

#### Significant Wave Height

Large waves also pose a risk to the mooring. A separate model exists for predicting wave activity in South East Tasmania, which is based on the Simulating Waves Nearshore (SWAN) model [19,20]. The mean significant wave height is calculated over the three months of model output available (January to March 2011), Figure 7.

### 3.4. Negotiation

Unfortunately not every location in public areas will be available for placing sensor nodes. There are some areas that will be completely off limits and some that will require some negotiation. Discussions with the relevant authorities will cost staff time in the planning stage and potentially in the future. In the TasMAN project we require permission from local and state government organisations in charge of the waterways. Their main concern is that we keep the sensor nodes out of shipping channels and high traffic areas to limit the impact on mariners.

To automate the cost of negotiation we used ENCs to find the shipping channels. If the shipping channels passes through a grid point it is given a high cost. To speed up the search process for deciding if a grid point is in a shipping channel, the shipping channels are stored in an R-tree structure [21].

**Figure 8.**
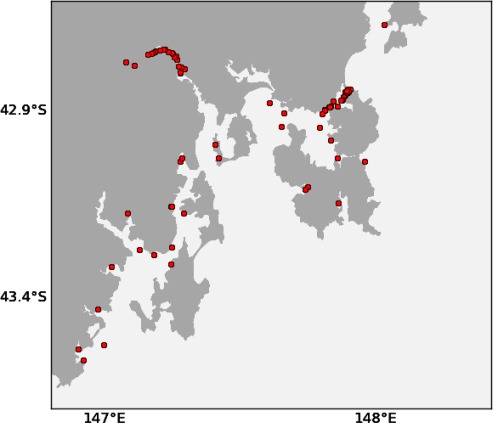
Locations of navigational aids in South East Tasmania.

We also give preference to locations that contain a navigational aid. The sensor node can be strapped onto the navigational aid causing less clutter and nuisance, as well as reducing mooring costs. The locations of navigational aids in South East Tasmania can be seen in Figure 8. These locations can also be derived from ENCs.

### 3.5. Communication

In more compact sensor networks, we may be able to reduce costs by communicating between nodes via radio and only use communications that incur costs, such as satellite or mobile telephone, from one node. There are existing techniques for designing networks for efficient communication [8,9] and these could be integrated at this stage.

The TasMAN low-cost sensor nodes are each equipped with their own 3G modems, mainly because of the large distances we are aiming to cover, so there is equal cost for every communication. We investigated the possibility that some areas might not have 3G coverage, but on inspecting the coverage map it did not appear to be an issue for South East Tasmania. The coverage map can be viewed at [22]. If this was not the case, we would have encoded the coverage map and attributed high cost to locations without adequate coverage.

## 4. Estimating Benefits

In some locations the additional cost will be worthwhile when it produces significant scientific gain. The gain will not be financial, but rather provide access to novel, highly variable, or relevant sensor information. We have identified several possible benefits:

- Interest to stakeholders
- High error areas
- Coverage

### 4.1. Interest to Stakeholders

There are multiple organisations who would like access to the data from our sensor network. Our stakeholders include; marine scientists, government agencies and industry. There will be areas that are of particular importance to these stakeholders. For example, areas that are good indicators of pollutant levels or of high uncertainty in the hydrodynamic model.

We set up a collaborative map that allows stakeholders to highlight areas of high interest. The resulting Keyhole Markup Language (KML) file is then parsed to create an R-tree structure [21], which we use to efficiently find the corresponding grid points in the hydrodynamic model [14]. Locations that lie within these high interest areas are given a high benefit score, and those that are not receive a benefit score of zero. The high interest areas can also be given priorities by attributing higher or lower benefit scores. These areas will usually overlap over many grid points and therefore the other cost and benefit scores will ultimately determine which is the best specific location within this area.

### 4.2. High Error Areas

Another way a location can improve the overall system is for it to be placed in a location that we are currently able to infer poorly. The score is based on the error at this location after we interpolate the existing nodes based on inverse distance. This error score will be updated as we add new nodes using the ACBA.

The error *e* at a particular grid point *g* is calculated over 100 random time steps *T* from two years of temperature and salinity data from the hydrodynamic model [14] using Equation (2). This is a standard method for calculating percentage error where *x* is the actual value, in this case taken from the model output for that grid point, and *y* is the estimated value, in this case the interpolated value from the three closest existing nodes.

(2) $$e(g)=\sum_{t\in T}\frac{\left|x_{t}-y_{t}\right|}{x_{t}}$$

The error map has to be built up from at least one node, which could be chosen from cost-benefit analysis initially without the error reduction benefit, or it could be chosen randomly. In our case, we use the existing nodes in the TasMAN network that were directly chosen by our stakeholders, and the glider transect.

**Figure 9.**
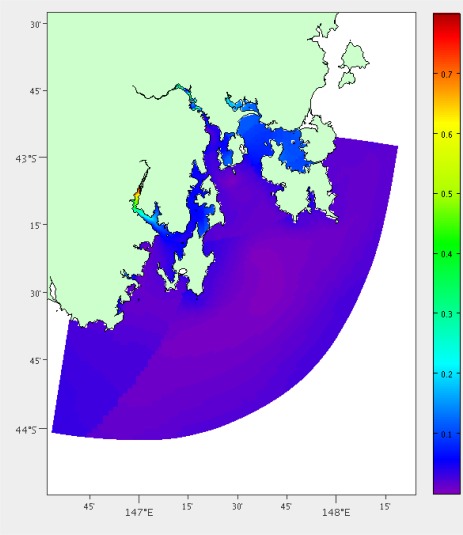
Percentage temperature and salinity error after interpolation using the hydrodynamic model for South-East Tasmania.

Figure 9 shows the areas of high error when we interpolate temperature readings from the existing three nodes. To the south west, we can see that upriver in the Huon (43°10′S, 147°E) is being represented the most poorly. This will increase the benefit of adding a node at this location. However, if we would like to add another node we recalculate these error scores. Placing a node should reduce the error in this area and reduce the likelihood of placing another there.

### 4.3. Coverage

In practice, we will always have limited sensor nodes available. If some locations are able to give us information about a large area then these should be also utilised, rather than concentrating solely on localised behaviours. The footprint of influence of a grid point is calculated by the number of grid points not yet covered that have error below a threshold. The error between two grid points being the difference between the values over time, as in Equation (2). Although in this case there is no interpolation as we are comparing a vector from two different locations. Again the data is 100 random time steps from two years of temperature and salinity data from the hydrodynamic model. In the case of these experiments, we set the threshold at 3%.

**Figure 10.**
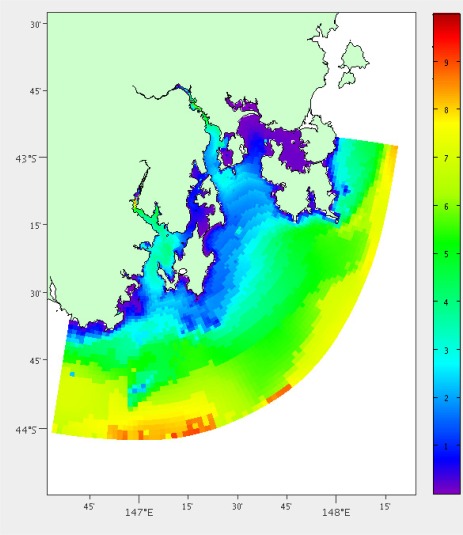
Temperature coefficient of variance across the depth profile.

We also compare the coefficient of variance over the depth profile. The coefficient of variance is calculated with *σ*/*μ* where *σ* is the standard deviation and *μ* is the mean. It is difficult to directly compare the other depths as they may be different from one location to another. In the case of South East Tasmania the depths range from 1 to 200 m. The difference between the coefficient of variance at the two locations must also be under a specified threshold (generally between 1 and 10%). The difference in coefficient of variance is gained using a method similar to Equation (2) over both temperature (Figure 10) and salinity.

The thresholds could potentially be derived from a relationship between the number of potential node locations and the number of sensor nodes you wish to deploy. For any additional nodes we need only recalculate the footprint size removing the grid points in the footprint of the added nodes. This takes negligible time and is scalable with high numbers of nodes to add.

**Figure 11.**
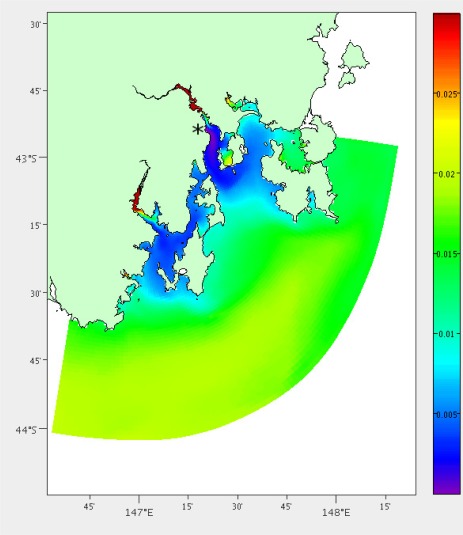
Influence of node at CSIRO Laboratory.

Figure 11 shows the footprint of influence of the location of our laboratory and one of our permanent sensor nodes. It is clear that closer to the location itself the stronger the similarity. However, we have noted that there is a reasonably strong similarity between the two major rivers, the Derwent (42°50′S, 147°15′E) and Huon (43°10′S, 147°E).

## 5. Cost-Benefit Analysis

To generate an overall cost for each location, we combine the costs *C*, which are each scaled to be between 0 and 1. For example:

C=[negotiation_cost, maintenance_cost,…]

We have chosen to add a weight *w* to each of the costs to allow more flexibility and customisation to a particular deployment. For example, in our first deployment of ten nodes, we are implementing new technologies so we would prefer to keep the nodes close to our laboratory. In other deployments, it may be more important to reduce the risk of damage and loss of sensor platforms. In this case, higher weight could be placed on the exposure scores.

**Figure 12.**
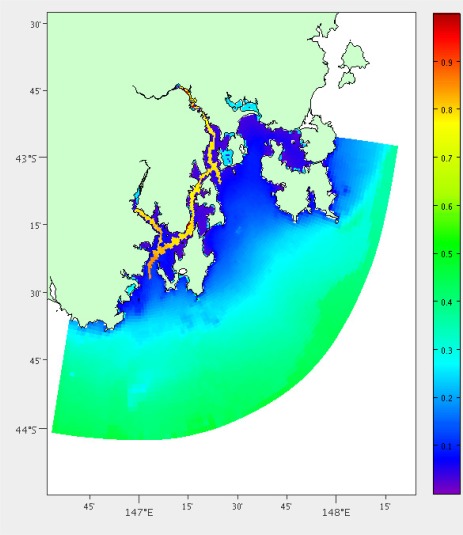
Combined cost scores for land distance, sea distance, depth, mean current velocity, wave height and shipping channels.

Combining the costs together gives us the map in Figure 12.

**Figure 13.**
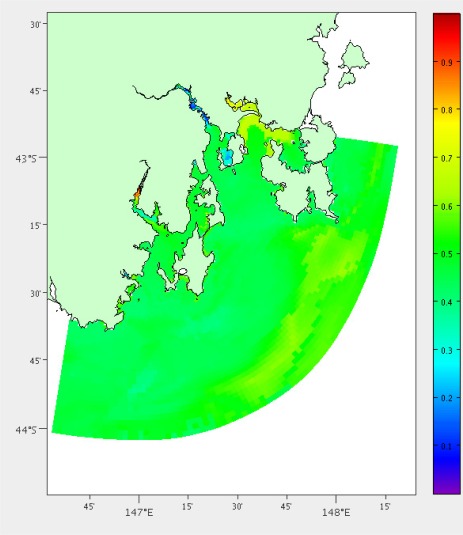
Combined benefit scores for coverage and error.

For the benefits *B*, a weight is also added to each benefit *b* so we can, for example, place emphasis on high interest areas for a particular deployment. The benefits for South East Tasmania can be visualised in Figure 13. This is the state of the combined benefits with additional weight on areas of interest when we first begin with the three existing nodes. The benefits will continue to change as we add new nodes. The highest cvb score can be seen around (43°10′S, 147°E) in Figure 13.

A cost *versus* benefit score *cvb* is then calculated for each potential location *l* using Equation (3).

(3) $$\mathrm{cvb}(l)=\frac{\sum_{n\in B}b_{n}\;w_{n}}{\sum_{n\in C}c_{n}\;w_{n}}$$

Before the benefits are divided by the costs the combined costs and benefits are scaled between 1 and 2.

To decide on where to place the next node, we simply chose the location with the highest *cvb* score. Then if we have more nodes to add we recalculate the footprint and error scores. As we only count in the footprint area locations that are not adequately covered by existing nodes this needs to be updated when we add a new node. We also need to perform the interpolation again for each location taking the new node into account before we update the error score. Once this has been performed we again find the location with the highest cost *versus* benefit score and repeat until we have run out of nodes to add.

Some pre-processing of cost and benefit values may be necessary as there is the possibility of very high costs skewing the normalisation and making it hard to differentiate between the low cost values that we are interested in. Some potential methods include: setting all cost values over a threshold to a maximum, or applying a quantisation function, such as binning.

Another approach might be to assign a cost “budget” to work within. In this case, the design system might choose to add many nodes with low cost, or to add fewer nodes in high cost high benefit areas. This is something we may investigate in the future, but is out of the scope of this paper.

**Figure 14.**
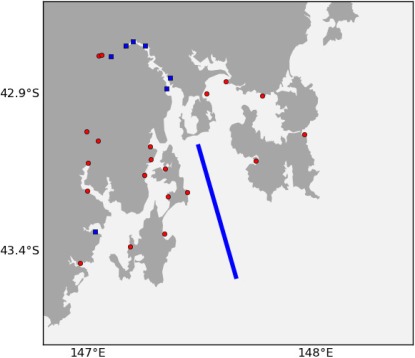
Locations of 20 additional nodes (red circles) in South East Tasmania using ACBA. The existing nodes are blue squares and a blue line represents the regular autonomous underwater vehicle transect.

**Figure 15.**
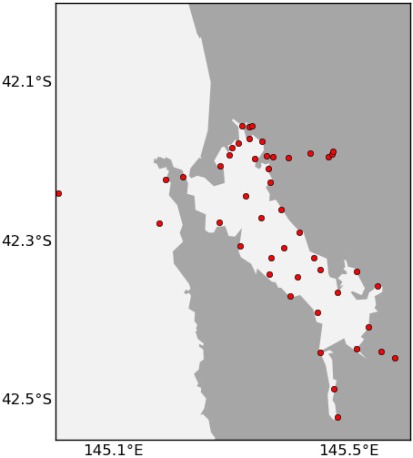
Locations of 50 additional nodes in Macquarie Harbour using ACBA.

The proposed node locations are then output in a KML file which is then uploaded as a collaborative map that we can send to the stakeholders of the sensor network. We can also send this link to the ports authority so they have time to properly evaluate the feasibility of the locations. The node markers are placed at the centre of the grid cell. Examples can be seen in Figures 14 and 15.

Within the KML file is an explanation of what weights were used and the contributing cost and benefit scores. This is important feedback that may inform weight adjustment, or the removal of contributing costs or benefits.

## 6. Results

**Figure 16.**
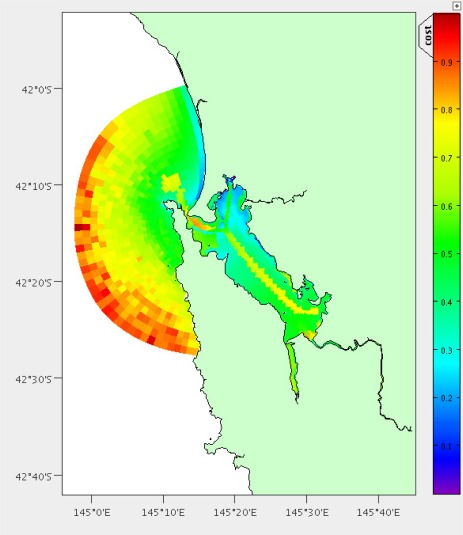
Estimated combined costs for Macquarie Harbour. The scale is in the bottom left (20km). Colour scale is in top right (Dark blue = 0 cost score, dark red = 1 cost score).

**Figure 17.**
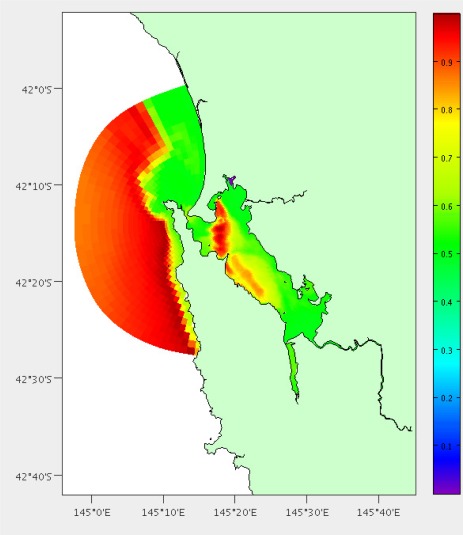
Estimated combined benefits for Macquarie Harbour. The scale is in the bottom left (20 km). Colour scale is in top right (Dark blue = 0 benefit score, dark red = 1 benefit score).

We have performed this analysis both on South-East Tasmania and Macquarie Harbour (Figures 15, 16 and 17) to demonstrate that this technique is relocatable. We may also decide to instrument this waterway on the West Coast of Tasmania in the future as it also has great potential for aquaculture.

The main boat ramp at the (only) town of Strahan (42.15°S, 145.31°E) was chosen as the base of operations and the hydrodynamic model is the same as described by Herzfeld [6] relocated to this location. There are 5838 potential locations using the grid points of the model and the area is approximately 40 km^2^. Macquarie Harbour is a very remote location with adequate roads only to the town of Strahan. Therefore we have chosen not to include distance by land in the costs. We also do not have areas of interest for stakeholders, so instead use the coverage and high error as benefits. All of the weights were set as 1; having the same effect as no weights. Figures 16 and 17 show the costs and benefits estimated for the Macquarie Harbour area.

Figure 14 shows the final results for using the ACBA method for adding 20 nodes in South East Tasmania without high interest areas. The method takes into account the six existing nodes and the regular transect performed by an autonomous underwater vehicle. Figure 15 shows the final results for the ACVB method for adding up to 50 nodes in Macquarie Harbour.

**Figure 18.**
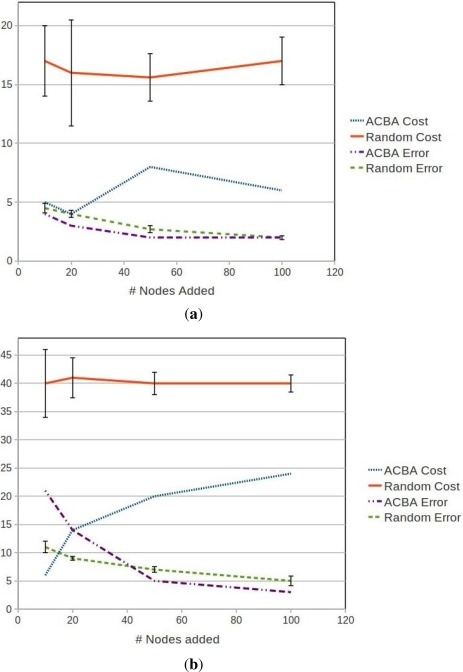
ACBA *versus* Random results for (**a**) South East Tasmania and (**b**) Macquarie Harbour. Cost values are in cost score arbitrary units and Error values are in percentage error.

Figures 18 contains the results from comparing the cost and benefit scores between our ACBA method and simply placing the nodes randomly. The ACBA method again had all weights set at one. Experiments were ran adding 10, 20, 50 and 100 nodes. The random cost and overall error were averaged over 10 runs and also began with the existing nodes in both regions. Once we have randomly selected the specified number of locations, the cost is determined by the combined costs we have quantified for that location. The overall error is calculated by the average of the difference between the interpolated values at each grid point and what the model actually forecast for that grid point. These were calculated using the separate test set.

**Figure 19.**
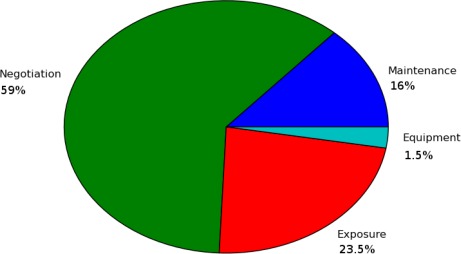
Cost structure at coordinates 42.9745°S, 147.762°E in South East Tasmania.

Figure 19 gives an example of a cost structure at 42.9745°S, 147.762°E in Norfolk Bay in South East Tasmania. This point lies on a shipping channel, so this makes up the greatest part of the cost for this location in the negotiation. The next biggest contribution is from the exposure, as there is a high current. It is also reasonably far from our laboratory giving it a fair cost in maintenance. It does not appear to be particularly deep or shallow at this location, so there is little impact from the equipment cost.

Outside of the TasMAN project, we have also used this technique to plan deployments of our low-cost nodes in Moreton Bay, Queensland (27.2°S, 153.2°E) following the floods of January 2011. This is a very busy shipping area, so we chose to place all of the sensor nodes on fixed infrastructure, in particular channel markers. The possible locations are therefore much more limited, but there were still hundreds of channel markers and only five nodes to deploy.

We have a collaborator that conducts regular manual water sampling surveys in Moreton Bay. The costs identified were the distance from the collaborators path, exposure (current), and customisation (depth). The benefits were in reduction of error and size of coverage. The data used was 100 random time steps over the period between September 2010 and February 2011, with 100 separate time steps reserved for testing.

**Figure 20.**
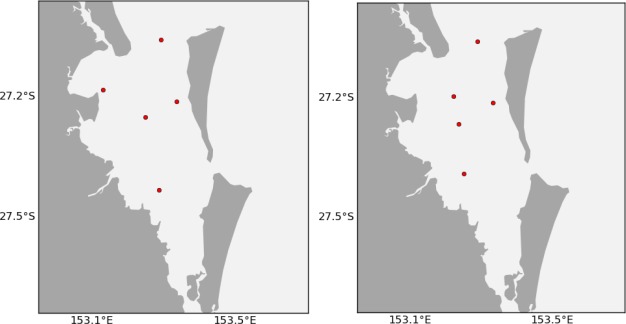
Locations of 5 nodes in Moreton Bay handpicked by a hydrodynamic model expert (left) and chosen using the ACBA method (right).

We asked a hydrodynamic modeller to pick some locations by hand. Figure 20 shows the expert-chosen locations and those chosen by ACBA. The locations are very similar, however the expert chosen locations have a combined cost score of 8.7 (average 1.74) and the ACBA locations have a combined score of 5.9 (average 1.18). The average error was calculated using a separate test set of 100 examples over the entire model area interpolated from the five chosen locations. The average error for the expert-chosen locations is 3.0% and the ACBA locations is 3.2%.

## 7. Discussion

There is a small improvement in error for South East Tasmania for all numbers of nodes, but it appears the Macquarie Harbour region is more difficult to cover at low cost. We are not able to produce equivalent error to random until we have more nodes. However, in both cases there is very large difference in estimated costs. The ACBA method always produces a configuration with much lower costs compared with every random configuration produced.

As expected, there is a point where adding additional nodes has limited effect on reducing the overall error. It is simple, using the current system, to specify an overall error threshold and keep adding nodes until you reach that threshold.

A limitation of using the hydrodynamic model is that we will not identify areas where the model does not match the observations, but until we have a greater granularity of observations from the sensor network, we cannot evaluate the accuracy of the model. This could be attempted on later deployments.

Using the grid format for the hydrodynamic model allows for easy visualisation using any tools that can display NetCDF files graphically. Viewing the costs in this way allows us to evaluate if we have missed anything relevant.

We have not considered that some locations would yield scientific gain from having one sensor, but not another. The temperature sensors we use are very inexpensive, the salinity sensors are slightly more expensive, but the dissolved oxygen and fluorometers have a much higher monetary cost. We have access to a biogeochemical model for this area, and are currently investigating integration with the current system to plan the deployment of fluorometers.

ACBA produced configurations with equivalent error to a hydrodynamic expert, but with lower estimated costs. This shows that this is a useful tool when experts are not available.

A modified version of ACBA has been implemented to plan cost-effective, high-scientific-gain mobile node missions. Experiments continue to be run with our autonomous surface vehicle.

## 8. Conclusion

The costs and benefits we have chosen to measure for marine sensing in these particular locations may need to be altered and customised for other locations and applications. However, we have aimed to demonstrate that the constraints and practicalities of sensor network deployment can be formalised to create an automated design system.

As the hydrodynamic model is relocatable and Electronic Navigational Charts are available for most populated areas, than this approach can easily be implemented at new locations.

The results presented suggest that using this automated cost-benefit analysis we can maintain equivalent representation of an area whilst dramatically reducing the financial costs involved.

## Figures and Tables

**Figure 7.**
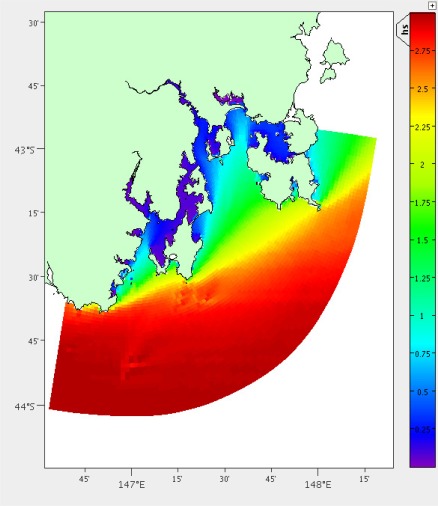
Mean significant wave height in metres for South East Tasmania from hydrodynamic model output.
